# Transcriptomic Analysis of a Diabetic Skin-Humanized Mouse Model Dissects Molecular Pathways Underlying the Delayed Wound Healing Response

**DOI:** 10.3390/genes12010047

**Published:** 2020-12-31

**Authors:** Carlos León, Francisco García-García, Sara Llames, Eva García-Pérez, Marta Carretero, María del Carmen Arriba, Joaquín Dopazo, Marcela del Río, María José Escámez, Lucía Martínez-Santamaría

**Affiliations:** 1 Department of Bioengineering, Carlos III University, Av. de la Universidad, 30, Leganés, 28911 Madrid, Spain; cleon@ing.uc3m.es (C.L.); MdelCarmen.Arriba@externos.ciemat.es (M.d.C.A.); mrnechae@ing.uc3m.es (M.d.R.); lmsantam@ing.uc3m.es (L.M.-S.); 2 Network Research on Rare Diseases (CIBERER), U714, C/Melchor Fernández Almagro, 3, 28029 Madrid, Spain; llamesccst@yahoo.es (S.L.); marta.carretero@ciemat.es (M.C.); 3 Regenerative Medicine and Tissue Engineering Group, Health Research Institute-Jiménez Díaz Foundation University Hospital (IIS-FJD), Av. de los Reyes Católicos, 2, 28040 Madrid, Spain; 4 Centre for Energy, Environment and Technology Research (CIEMAT), Av. Complutense 40, 28040 Madrid, Spain; 5 Bioinformatics and Biostatistics Unit, Prince Felipe Research Center (CIPF), C/Eduardo Primo Yúfera, 3, 46012 Valencia, Spain; fgarcia@cipf.es; 6 Spanish National Bioinformatics Institute, ELIXIR-Spain (INB, ELIXIR-ES), C/Eduardo Primo Yúfera, 3, 46012 Valencia, Spain; 7 Tissue Engineering Unit, Blood and Tissue Community Center of Asturias (CCST), C/Emilio Rodríguez Vigil s/n, 33006 Oviedo, Spain; evagp2002@yahoo.es; 8 Clinical Bioinformatics Research Area, Progress and Health Foundation (FPS), CDCA, Hospital Virgen del Rocío, Av. Manuel Siurot s/n, 41013 Sevilla, Spain; joaquin.dopazo@juntadeandalucia.es; 9 Functional Genomics Node, INB-ELIXIR-es, Progress and Health Foundation (FPS), Hospital Virgen del Rocío, Av. Manuel Siurot s/n, 41013 Sevilla, Spain; 10 Institute of Biomedicine of Seville (IBIS), Hospital Virgen del Rocio, Av. Manuel Siurot s/n, 41013 Sevilla, Spain; 11 Servicio de Análisis de Sistemas de Información Sanitaria, Consellería de Sanitat Universal i Salut Pública, Generalitat Valenciana, Carrer de Misser Mascó, 31-33, 46010 Valencia, Spain

**Keywords:** transcriptomics, wound healing, diabetes, skin-humanized mice, enrichment analysis

## Abstract

Defective healing leading to cutaneous ulcer formation is one of the most feared complications of diabetes due to its consequences on patients’ quality of life and on the healthcare system. A more in-depth analysis of the underlying molecular pathophysiology is required to develop effective healing-promoting therapies for those patients. Major architectural and functional differences with human epidermis limit extrapolation of results coming from rodents and other small mammal-healing models. Therefore, the search for reliable humanized models has become mandatory. Previously, we developed a diabetes-induced delayed humanized wound healing model that faithfully recapitulated the major histological features of such skin repair-deficient condition. Herein, we present the results of a transcriptomic and functional enrichment analysis followed by a mechanistic analysis performed in such humanized wound healing model. The deregulation of genes implicated in functions such as angiogenesis, apoptosis, and inflammatory signaling processes were evidenced, confirming published data in diabetic patients that in fact might also underlie some of the histological features previously reported in the delayed skin-humanized healing model. Altogether, these molecular findings support the utility of such preclinical model as a valuable tool to gain insight into the molecular basis of the delayed diabetic healing with potential impact in the translational medicine field.

## 1. Introduction

Diabetes is a systemic chronic disorder with a high and continuously increasing incidence and its global prevalence is projected to reach 10.2% by 2030 [1]. Impaired wound healing is one of the major complications associated to diabetes; in fact, between 19% and 34% of diabetic patients will develop a foot ulcer during the course of their illness [2]. High ulcer recurrence rates as well as frequent infections contribute to prolonged hospitalization and lead to an increased risk of lower limb amputation which has significant effects on patient morbidity and mortality [2]. Consequently, the quality of life of patients is often compromised. Furthermore, high costs associated with the clinical management of recurrent diabetic wounds represent a significant economic impact on the healthcare system. 

A persistent inflammatory response, impaired fibroblast function, hyperproliferative non-migratory epidermis, decreased angiogenesis, altered extracellular matrix (ECM) deposition, increased levels of proteases, and unresponsiveness to growth factor signals are the most recognized features of non-healing diabetic wounds [3,4,5,6]. However, the incomplete understanding of the underlying mechanisms responsible for defective diabetic healing contributes to the lack of effective treatments [7]. The study of diabetic wound healing in patients is limited mainly by technical and ethical considerations as well as by the complexity and heterogeneity of the disease. For these reasons, a plethora of murine models of diabetes have been developed [8,9,10] with the consequent flaws in extrapolation of the results due to functional and structural differences between human and rodent models. On the other hand, studies in large animals such as pigs are troublesome and expensive. In this context, the use of humanized mouse models poses a great potential for mimicking human conditions more accurately while preserving some of the advantages of the use of animal experimentation. 

Our group has extensive experience in generating humanized models based on the permanent engraftment of human bioengineered skin onto the back of immunodeficient mice [11,12,13,14] that in fact resemble a wide variety of physiological and pathological cutaneous processes, including wound healing [15,16]. Specifically, we developed a diabetes-induced delayed humanized wound healing model that reproduced some of the main cutaneous features described in the healing impairment of diabetic patients, including a prolonged inflammatory response, poor dermal matrix remodeling, impaired angiogenic response, and impaired migratory activity of keratinocytes [17]. These humanized models have also been suitable platforms to test pharmacologic, cell and gene therapeutic approaches [13,15,16,17,18].

A broad variety of analytical tools including standard cell, molecular, and different “omics” technologies have been used for unravelling deregulated processes in wound healing [19,20,21]. In the present work, a transcriptomic study together with a functional enrichment analysis of the wound healing response, followed by a more sophisticated mechanistic signaling pathway analysis [22,23], were performed in the diabetic skin-humanized mouse model aiming to compare our results with previous studies in diabetic patients and in such preclinical model. 

## 2. Materials and Methods 

### 2.1. Wound Healing Experimental Design in the Diabetic Skin Humanized Mouse Model

NMRI nude mice (Rj:NMRI-Foxn1nu; Elevage Janvier Laboratories, Le Genest Saint Isle, France) were orthotopically grafted with bioengineered cutaneous equivalents [11,12]. This skin equivalent is based on human keratinocytes (epidermal component) seeded onto the top of the fibrin matrix populated with live human fibroblasts (dermal component). Cells from a skin biopsy of a healthy donor were isolated as previously described [11,12,24] after informed written consent and in accordance with the Helsinki declaration of 1975 and further revisions and with the Spanish regulation. The grafting of bioengineered human skin equivalents was performed under sterile conditions at the Centro de Investigaciones Energéticas Medioambientales y Tecnológicas (CIEMAT) Laboratory Animals Facility (European registration number ES280790000183). All experimental procedures were performed in accordance with the corresponding regulations regarding experimental animal welfare. Experimental diabetes was induced in skin-humanized mice 10 weeks postgrafting by intraperitoneal injections of streptozotocin (STZ; Sigma-Aldrich, St. Luis, MO, USA), as previously described [17]. Wound healing experiments were performed in the stable human skin engrafted on immunodeficient mice exposed to sustained hyperglycemia for 6 weeks. Specifically, 2 mm-circular excisional wounds were created, and the excised tissue was harvested and used as a reference for gene expression analysis. After 24 h, a 6 mm-circular ring around the wound edge was taken. Samples were transferred immediately into liquid nitrogen for fast freezing and stored at −80 °C until processing. A total of 26 skin samples were obtained (*n* = 7 for control mice and *n* = 6 for diabetic mice for each condition, i.e., t = 0 h and t = 24 h).

### 2.2. RNA Extraction

Total RNA isolation was performed using TRIzol^®^ (Invitrogen, Carlsbad, CA, USA) extraction-based methods, followed by purification in columns (Qiagen, Hilden, Germany). Briefly, skin samples were completely lysed in 1 mL of TRIzol, and subsequently 0.2 mL of chloroform was added to the suspension. After incubation at room temperature for 5 min to allow phase separation, the mixture was centrifuged at 14,000 rpm (15 min at 4 °C) and RNA was isolated from the aqueous phase. RNA was then precipitated with isopropanol, washed with 70% ethanol, and resuspended in RNase-free water (Qiagen). Total isolated RNA was further purified with the RNeasy kit (Qiagen). RNA concentration was determined using a NanoDrop™. Spectrophotometer (Thermo Scientific, Waltham, MA, USA) and RNA integrity was verified with a Bioanalyzer (Agilent, Santa Clara, CA, USA).

### 2.3. Microarray Analysis

Total RNA extracted from 26 skin samples was used to generate double-stranded cDNA. Briefly, biotin-labeled cRNA was fragmented and hybridized overnight to an Affymetrix Human Genome GeneChip^®^ (HU133 2.0 GeneChip, 22277 probesets), according to the manufacturer’s protocol. After 16 h of hybridization at 45 °C, arrays were washed, stained with streptavidin-phycoerythrin, and then scanned with the GeneArray Scanner (Affymetrix, Santa Clara, CA, USA). The quality control of the scanned images included the examination of visible artifacts, the confirmation of proper grid alignment and the subtraction of background intensity. GeneChip^®^ operating software (GCOS) was used to generate “.CEL” files. 

All raw data comply with the Minimum Information About a Microarray Experiment (MIAME) guidelines. Gene expression DataSets are available in the Gene Expression Omnibus (GEO) on the NCBI website (http://www.ncbi.nlm.nih.gov/geo; accession number GSE147890).

### 2.4. Data Processing and Statistical Analysis

Raw data from microarrays was normalized using the rma algorithm from the affy package (Bioconductor 3.12 Released) [25]. This algorithm includes background correction, quantile normalization and probeset summarization using the median. A Principal Component Analysis (PCA) was performed to the normalized data in order to detect the presence of outliers. Differential expression analysis between groups was evaluated with the limma package from Bioconductor [26] using the Babelomics suite v5.0 [27]. The *p*-values were corrected using the Benjamini–Hochberg method for multiple testing and FDR [28]. Finally, a functional enrichment analysis was performed using the updated versions of Gene Ontology [29] and the Kyoto Encyclopedia of Genes and Genomes (KEGG) [30] annotation databases through the tool incorporated in the Database for Annotation, Visualization, and Integrated Discovery (DAVID v6.8) [31].

### 2.5. Pathway Activity Analysis

A signaling circuit activity analysis method implemented in the Hipathia tool [23] was employed to study wound healing in both control and diabetic mice. Under this approach, signaling circuits are defined within KEGG signaling pathways as the chain of proteins that connect a receptor protein to effector proteins that trigger specific cellular activities. Normalized gene expression values are taken as proxies of protein activity.

## 3. Results

### 3.1. Exploratory Microarray Data Analysis

**Figure 1:**
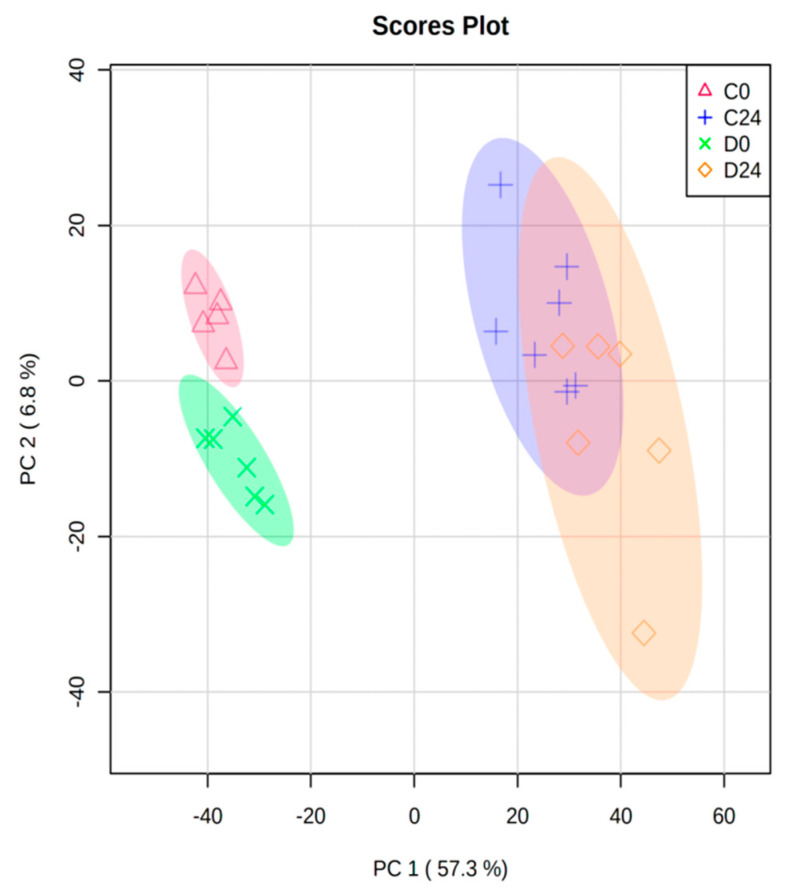
Principal Component Analysis (PCA) biplot of all normalized samples (*n* = 24) from the 4 groups (C0: control samples at 0 h; C24: control samples at 24 h; D0: diabetic samples at 0 h; D24: diabetic samples at 24 h). Circles show 95% confidence.

RNA extracts of 26 skin-humanized samples were hybridized to Affymetrix GeneChip^®^ Human Genome U133 2.0 Array. The total expression of >20,000 probes was normalized and preprocessed. The normalized data for all probes in all samples is provided in Table S1. First, in order to ensure the highest data quality, a PCA was performed. Two control samples taken at 0 h were found as outliers (95% confidence) and discarded to avoid technical variability bias in the data analysis. The remaining 24 samples were clearly grouped into 4 different categories: control samples at 0 h and 24 h, and diabetic samples at 0 h and 24 h (Figure 1). Specifically, the bigger difference in gene expression between groups was related to wound healing process (0 h versus 24 h). In fact, PC1 mainly separated samples from 0 h and 24 h (57.3% variance) while PC2 (6.8% variance) was the main factor discriminating between the diabetic and control mice (Figure 1).

Differential expression analysis was performed using limma package from Bioconductor. As in the PCA analysis, the number of differentially expressed (DE) probes indicated that the main driving force in terms of differential gene expression was mainly triggered in response to the cutaneous wound during the first 24 h (Table 1). Indeed, and since the experimental diabetes process was sustained for a longer period (6 weeks), a minor number of genes was affected at the time of sampling. 

### 3.2. Differential Expression and Functional Enrichment Analysis of the Diabetes-Induced Skin-Humanized Mouse Model

In relation to the experimentally induced diabetes in the skin-humanized mouse model, 403 probes were differentially expressed (FDR < 0.05) between both groups of animals before wounding (D0 vs. C0), mainly in response to the diabetes induction. Likewise, the number of biological processes (GO_BPs) and KEGG pathways associated with those dysregulated probes followed the same trend (Table 1). However, the number of DE probes between diabetic and control mice at 24 h (D24 vs. C24) was much lower (49 probes). This could be explained due to the bigger variability of samples taken at 24 h compared to the samples at 0 h which in fact, formed a more compact cluster (Figure 1). 

**Figure 2:**
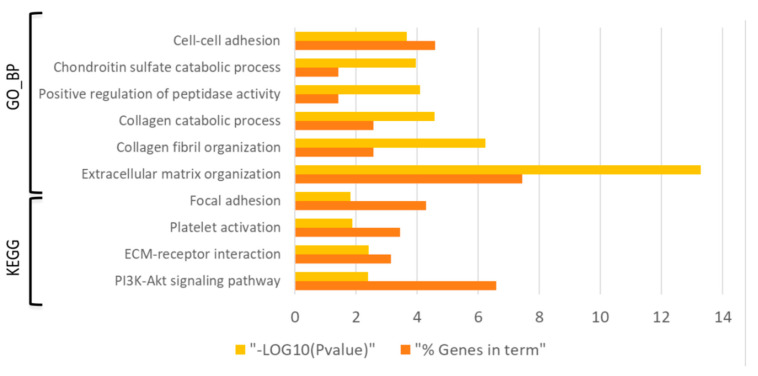
Bar graph showing the dysregulated Gene Ontology biological processes (GO_BPs) and KEGG pathways (FDR < 0.05) with the lowest *p*-value (*p* ˂ 0.01) in the diabetes induction process (D0 vs C0 comparison). Yellow bars indicate the *p*-value of the term in log form and orange bars indicate the percentage of dysregulated genes in each term.

Enrichment analysis of those 403 DE probes using DAVID bioinformatics tool showed a total of 100 biological processes from the Gene Ontology and 7 KEGG pathways (FDR < 0.05) associated to the gene expression changes during the process of experimental diabetes induction (i.e., D0 vs C0 comparison) (Table S3). Specifically, terms such as ECM organization, cell-cell adhesion as well as PI3K/Akt signaling, ECM receptor interaction and focal adhesion were altered in the diabetes induction process (Figure 2).

### 3.3. Differential Expression and Functional Enrichment Analysis of the Wound Healing Process in the Diabetes-Induced Skin-Humanized Mouse Model

**Figure 3:**
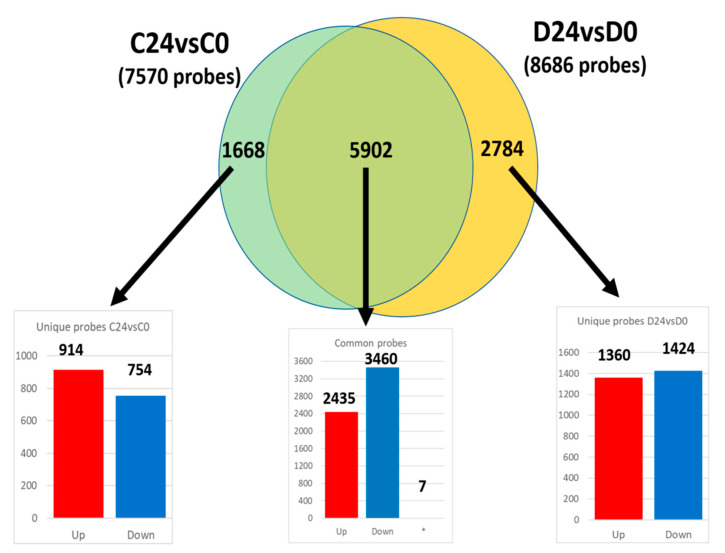
Venn diagram of common and unique differentially expressed probes in both wound healing processes (C24vsC0 and D24vsD0). The number in each bar graph indicates the up-(red) and down-regulated (blue) probes (FDR < 0.05). (*): Seven probesets out of the total 5902 common ones showed opposite regulation.

Aiming to study the functional similarities and differences in both wound healing processes (C24vsC0 and D24vsD0), two enrichment analyses were carried out. Specifically, the common dysregulated probes and also the specific probes to each process were identified (Figure 3).

#### 3.3.1. Common Transcriptomic Response to Wound Healing in Both Control and Diabetic Mice

Out of the 7570 and 8686 probes dysregulated in the wound healing process of control and diabetic mice respectively (C24vsC0 and D24vsD0; Figure 3), 5902 probes were common in both groups, and therefore linked to the same dysregulated functions and pathways. The fold change of these probes was also surprisingly similar in both comparisons (Figure S1A), displaying a strong linear correlation (slope = 1.074; R2 = 0.93). However, seven probes out of those 5902 had a different expression pattern between groups (i.e., upregulated in controls and downregulated in diabetic animals, or vice versa) (Figure S1B) which suggests an opposite mechanism in the functions related to those seven genes in the different experimental groups. 

**Figure 4:**
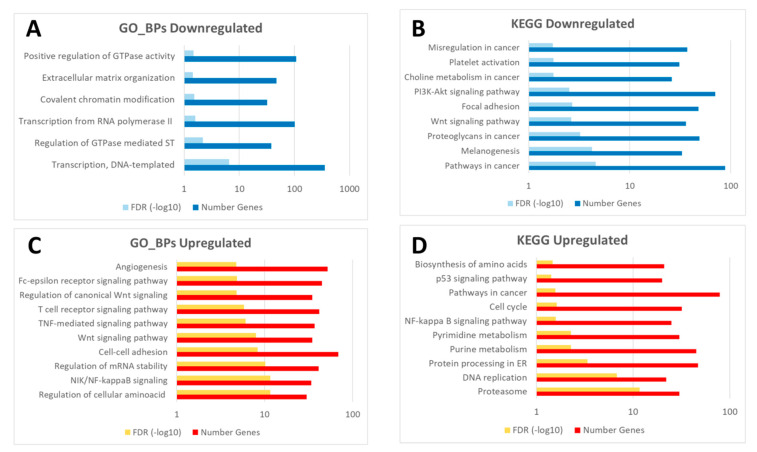
Diagram of common enriched functions (GO_BPs: Gene Ontology biological processes, and KEGG pathways) in the downregulated (**A**,**B**) and upregulated probes (**C**,**D**) in response to wounding in both groups (C24vsC0 and D24vsD0). The complete list of enriched GO_BPs and KEGG pathways is available in Table S4 (sheet tabs A and B).

An enrichment analysis of the common transcriptomic response to wounding (5895 probes displaying the same expression pattern) in both control and diabetic mice was carried out. Thus, upregulated and downregulated probes at 24 h post-wounding (2435 and 3460 probes respectively; Figure 3) were analyzed separately. Specifically, downregulated genes were predominantly enriched in functions related to transcription processes, gene regulation and ECM organization (Figure 4A,B). On the other hand, upregulated probes were enriched in a very wide array of functions, many of them related to the inflammatory response after wounding, such as Wnt signaling pathway, NFkβ signaling, TNF mediated response, as well as DNA replication, cell–cell adhesion or MAPK signaling pathways and angiogenesis, among others (Figure 4C,D).

#### 3.3.2. Different Transcriptomic Response to Wound Healing in Control and Diabetic Mice

A functional enrichment analysis of the probes which altered expression was unique to either control (1668) or diabetic healing (2784) (Figure 3), was performed (Table 2). Specifically, upregulated and downregulated probes were studied separately, aiming to understand the wound healing process inherent to each experimental group. 

Interestingly, a marked enrichment for GO_BPs and KEGG pathways (204 and 72 functions, respectively) was observed in upregulated probes in the diabetic wound healing process (unique in D24vsD0). Specifically, pathways related to cellular movement and response to mechanical stimulus as well as to the inflammatory response, such as TGFβ, NFkβ, TNF, and chemokine signaling pathways, were enriched in those probes (Table S4C). There were also many unique GO_BPs related to deregulation of apoptosis processes. 

On the other hand, unique pathways in the control wound healing process (C24vsC0) were mainly related to mitochondrial and oxidative phosphorylation processes (upregulated genes), as well as to transcriptional regulation (downregulated genes) (Table S4, sheet tabs E and F).

**Figure 5:**
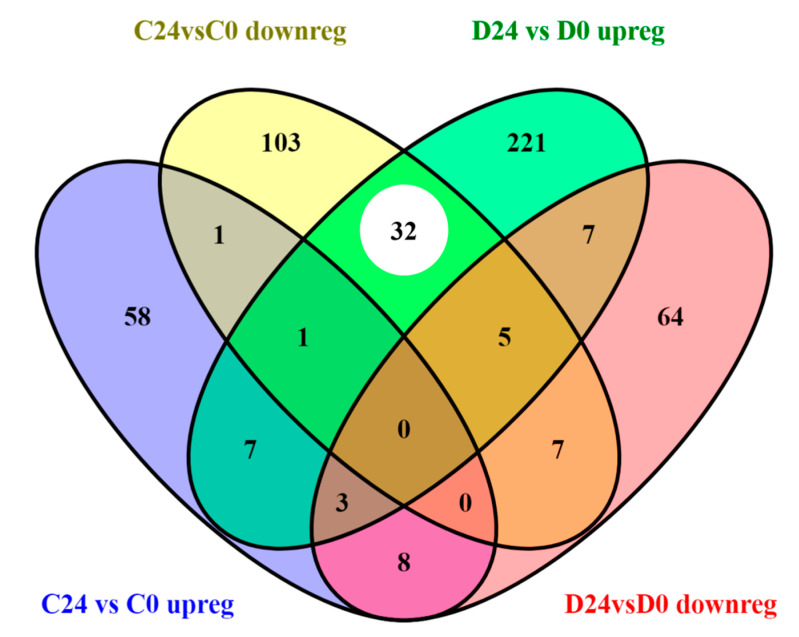
Venn diagram of the number of functions (Gene Ontology biological processes and KEGG pathways) altered in unique probes up and downregulated in C24vsC0 (1668 DE probes) and D24vsD0 (2784 DE probes) groups, respectively. White circle shows coincident functions altered by genes with opposite regulation (upregulated in D24vsD0 and downregulated in C24vsC0).

Aiming to identify processes equally altered in both wound healing processes but due to the dysregulation of different genes, coincident and discordant functions enriched in unique probes in C24vsC0 and D24vsD0 were evaluated (Figure 5). 

It must be noted that the number of coincident pathways was significantly higher between upregulated genes in the diabetic wound healing and the downregulated ones in control samples (as indicated by a white circle in Figure 5 and highlighted in yellow in Table S4). This fact suggests that several functions or metabolic routes were being triggered in opposite directions in response to wound healing in both experimental groups, including regulation of cell proliferation, fibroblast growth factor signaling pathways, FoxO signaling pathway, TFGβ receptor signaling pathway, cell migration, and ECM organization, among others (Table S4). Some of these functions have been already associated to impaired diabetic healing [5,32,33].

### 3.4. Mechanistic Signaling Pathway Analysis of the Wound Healing 

To further describe the underlying molecular mechanisms involved in the wound healing process, a mechanistic activity analysis that decomposes KEGG signaling pathways into canonical sub-pathways (circuits) was employed. This method connects receptor proteins, by means of a chain of intermediate proteins, to effector proteins that trigger specific cell activities. This analysis was performed using the normalized expression values of both C24vsC0 and D24vsD0 comparisons. All significant circuits (FDR < 0.05) and effector proteins are included in Table S5. Specifically, a total of 382 circuits involved in 53 KEGG pathways (D24vsD0) and 424 circuits in 55 pathways (C24vsC0) were affected in each comparison. The analysis of the common and unique altered circuits evidenced that most of them (262 circuits) were common to both wound healing processes while only 102 and 137 circuits were unique in D24vsD0 and C24vsC0, respectively. In both cases, these circuits were mainly involved in KEGG pathways already highlighted in the enrichment analysis such as TGFβ, TNF, NFkβ, PI3K, Wnt, and FoxO signaling pathways, as well as apoptotic processes. In addition, there was a total coincidence in the direction (up or downregulation) of the common circuits between both comparisons. 

## 4. Discussion

There is still a lack of understanding of the underlying diabetes wound repair impairment. Since the use of human samples has ethical and technical issues that cannot be easily ignored [21], different animal models of diabetes have been developed in the last decades [8,9,10]. Although a huge amount of valuable basic knowledge has been obtained from such small pre-clinical animal models, they do not totally reproduce the complexity of the human disease, thus it is mandatory to recreate humanized contexts aiming to shed light on the molecular and cellular mechanisms responsible for diabetes–associated impaired healing. The humanized mouse model developed by our group [11,12,24] has been proven as a suitable platform to perform wound-healing studies in a humanized context since all major features of human cutaneous wound healing are accurately recapitulated [15,16,17]. In addition, we have developed humanized models that recreate a wide range of human cutaneous disorders in which interactions between the immune system, epidermis and the environment likely occur such as psoriasis and different genodermatosis [13]. Currently, humanized mice carrying both a functioning immune system and skin of human origin are being developed aimed to better recapitulate human cutaneous physiology [34]. These multi-tissue compartment models, though promising, need further research. Herein, we present the transcriptomic analysis of a humanized model of delayed wound healing in a diabetic context in order to dissect the early molecular mechanisms underlying diabetes wound repair impairment in such preclinical model. Since the major transcriptomic changes are usually observed within the first 24 h after wounding [35,36], the samples were taken at this time point. An enrichment and a mechanistic pathway analysis were also performed using the Gene Ontology and KEGG databases, thus the comparison was done at a functional level, and not only based in lists of dysregulated genes. Furthermore, this approach also allowed us to compare biological processes or pathways that may be equally altered in both diabetic and control wound healing processes, although through different gene expression variations.

The analysis of the transcriptomic data in the chemically induced diabetic skin-humanized mouse model showed that there were more differences in the gene expression due to the diabetes induction at the basal level (D0 vs. C0) than 24 h after wounding (D24 vs. C24). Indeed, this observation might be due to an acute genetic response to healing at 24 h in both experimental groups that would be drastically modifying the global gene expression and thus, minimizing the intrinsic differences between diabetic and control mice. This fact could also explain that a minor number of probes were deregulated in unwounded skin samples (D0 vs. C0) than in both healing processes at 24 h (D24 vs. D0 and C24 vs. C0).

In general terms, the differential global gene expression in the skin of the diabetic humanized mice (D0vsC0) closely reproduced the cutaneous transcriptome profiling in diabetic patients [37,38]. Specifically, those studies showed altered GO_BPs and KEGG pathways related to focal adhesions, cell adhesions mediated by integrins, and ECM-related processes, as in our diabetic skin-humanized mouse model (Figure 2). In addition, other relevant functions such as PI3K/Akt signaling pathway and collagen deposition mechanisms reported in diabetic patients [39,40] were also identified in our humanized model. Nevertheless, it must be noted that, unlike long-term exposure to hyperglycemia in diabetic patients, samples in the diabetic skin-humanized mouse model were taken after six weeks of sustained high glucose levels. Therefore, other deregulated molecular mechanisms triggered later by longer exposition to hyperglycemia might also be involved. Notwithstanding this fact, the main cutaneous features reported in diabetic patients, such as decreased innervation and vascularization, were faithfully mimicked in the regenerated human skin of diabetic mice after six weeks of sustained high glucose levels [17]. 

Regarding wound healing, an almost perfect linear correlation (R2 = 0.93) of the fold changes of common deregulated probes (5902; Figure 3) was evidenced between both experimental groups (C24vsC0 and D24vsD0; Figure S1A). However, only seven genes showed opposite regulation (i.e., upregulated in C24vsC0 and downregulated in D24vsD0 or vice versa) (Figure S1B). Those seven genes were related to functions such as cell proliferation (NDRG2 and TTK), cell adhesion (TRO and PLEC1) or inflammatory signaling processes (RIOK3 and JAG1) in accordance with previous findings in diabetic patients [3,41]. Remarkably, these deregulated molecular mechanisms might also underlie some of the histological features previously identified in our delayed skin-humanized wound healing model, such as a prolonged inflammatory response [17].

A deeper study of the common GO_BPs and KEGG pathways to both wound healing processes (Figure 4) showed the deregulation of functions related to the tissue repair process such as angiogenesis and TNF-mediated, Wnt, and NFkβ signaling pathways [42,43]. All these pathways were also identified in the mechanistic approach including the proposed effector proteins in each circuit (Table S5). Furthermore, VEGF-, SDF-1- and matrix metalloproteinases-related genes, critical factors during wound repair [5], were significantly upregulated in both comparisons (C24vsC0 and D24vsD0), also displaying a high fold change (Table S2). Specifically, the upregulation of VEGF in the VEGF signaling pathway starts a cascade response that involves the dysregulation of circuits related to cell adhesion, angiogenesis and lipid metabolism through the effector proteins PXN, PTK2 and PTGS2, respectively (Figure S2). Although some of these proteins have been identified under diabetic conditions [44], their role in wound healing should be deciphered.

Finally, the biological processes specifically altered in wound healing response in the physiological or diabetic condition (i.e., those probes that were unique in either C24vsC0 or D24vsD0) were identified (Table S4). Remarkably, terms related to inflammatory processes, such as TGFβ, FoxO, NFkβ, and TNF and chemokine signaling pathways, were particularly significant and abundant in the upregulated probes during the diabetic healing response (D24vsD0). These pathways, also identified in diabetic patients [32,33,45], might explain the prolonged persistence of neutrophils observed in the delayed humanized wound healing model [17]. In addition, the upregulation of inflammatory processes in chronic wounds has been previously linked with alterations in the apoptosis pattern [46]. Likewise, a high abundance of GO_BPs terms related to apoptosis was evidenced in the wounds of diabetic skin-humanized mice. Interestingly, both inflammatory and apoptotic processes were also evidenced in the mechanistic analysis approach, providing a more detailed insight into possible effector proteins that trigger specific cellular activities for all these pathways. Finally, other process altered in D24vsD0, such as the negative regulation of EGFR signaling pathway, could be also hampering the tissue repair progress, since impaired EGFR signaling have been identified in diabetic ulcers and in fact, treatments that restore such pathway have a healing potential for those patients [47,48].

Interestingly, a significant number of terms between both experimental groups showed opposite regulation (Figure 5). Remarkably, a high coincidence of altered functions due to downregulated genes in C24vsC0 but upregulated in D24vsD0, previously linked to impaired diabetic cutaneous wound healing [5,32,33], was observed. Those terms, responding in different directions in the physiological and the diabetic conditions, included the negative regulation of cell proliferation, the positive regulation of NFkβ transcription factor activity as well as TGFβ, MAPK, fibroblast growth factor receptor and FoxO signaling pathways. As an interesting remark, it has been demonstrated that FoxO signaling pathway has opposite effects on normal and diabetic healing and in fact, its deregulation has been proposed as a mechanism responsible of impaired tissue repair [49]. In fact, the role of FoxO signaling pathway should be further elucidated in our model. 

In conclusion, we performed an in-depth transcriptomic study by using a mechanistic approach that query global changes in diabetic wound tissue in a well-recreated humanized context. The transcriptomic profile was performed in an experimentally induced diabetes skin-humanized model. The molecular response was analyzed at 24 h after wounding aimed to identify the early healing response. Indeed, further studies at different time points will provide a full characterization of key pathways during the final stages of diabetic healing. Also, future research should include the use of next generation sequencing technologies aimed to provide a wider comprehensive analysis including genes with low expression as well as novel transcripts. Nevertheless, some of the transcriptional mechanisms also impaired in diabetic patients were faithfully replicated in the delayed humanized wound healing model. In fact, these molecular findings confirm the usefulness of the humanized preclinical model as a valuable tool to dissect the molecular pathways underlying the healing impairment and therefore to improve the design of meaningful therapies with a potential clinical impact.

## Figures and Tables

**Table 1:** Number of probes differentially regulated (FDR < 0.05) in each group comparison and their corresponding enriched Gene Ontology Biological Processes (GO_BPs) and KEGG pathways. All the differentially expressed probes in each comparison together with their *p*-value and fold change can be found in Table S2.

Comparison	Differentially Expressed Probes	Up-Regulated Probes	Down-Regulated Probes	Significant GO_BPs	Significant KEGG
C24 vs. C0	7570	3350	4220	456	42
D0 vs. C0	403	101	302	100	7
D24 vs. C24	49	14	35	3	0
D24 vs. D0	8686	3801	4885	519	71

**Table 2:** Number of significant Gene Ontology biological processes (GO_BPs) and KEGG pathways enriched in the unique up- and down-regulated probes (FDR < 0.05) in both C24vsC0 and D24vsD0 comparisons. The list of the significant terms can be found in Table S4 (sheet tabs C–F).

Comparison	Significant GO_BPs		Significant KEGG	
	Up-Regulated Probes	Down-Regulated Probes	Up-Regulated Probes	Down-Regulated Probes
Unique C24vsC0	67	122	11	27
Unique D24vsD0	204	86	72	8

## Data Availability

Gene expression DataSets are available in the Gene Expression Omnibus (GEO) on the NCBI website (http://www.ncbi.nlm.nih.gov/geo; accession number GSE147890).

## Supplementary Materials

The following are available online at https://www.mdpi.com/2073-4425/12/1/47/s1, Figure S1. (A) Graphical representation of the fold change of common regulated genes in both wound healing processes (C24vsC0 and D24vsD0). (B) Diagram of the fold change of the seven genes with opposite regulation in C24vsC0 (blue) and D24vsD0 (red). Figure S2. VEGF signaling pathway identified in the mechanistic approach. VEGF upregulation triggers a cascade of changes in cellular activities such as cell adhesion, angiogenesis or apoptosis. Blue nodes: downregulated genes; red nodes: upregulated genes. White boxes: cellular activities altered by an effector protein. Table S1: Normalized gene expression data. Table S2: Differentially expressed genes in all comparisons. Table S3: Enrichment analysis in the diabetes induction process. Table S4: Enrichment analysis in the wound healing process. Table S5: Mechanistic analysis in the wound healing process.
