# Supplementary figures and images for: Transcriptomic Analysis of a Diabetic Skin-Humanized Mouse Model Dissects Molecular Pathways Underlying the Delayed Wound Healing Response
Source: Genes (Basel). 2020 Dec 31;12(1):47. doi: 10.3390/genes12010047 (PMC7824036; doi:10.3390/genes12010047)

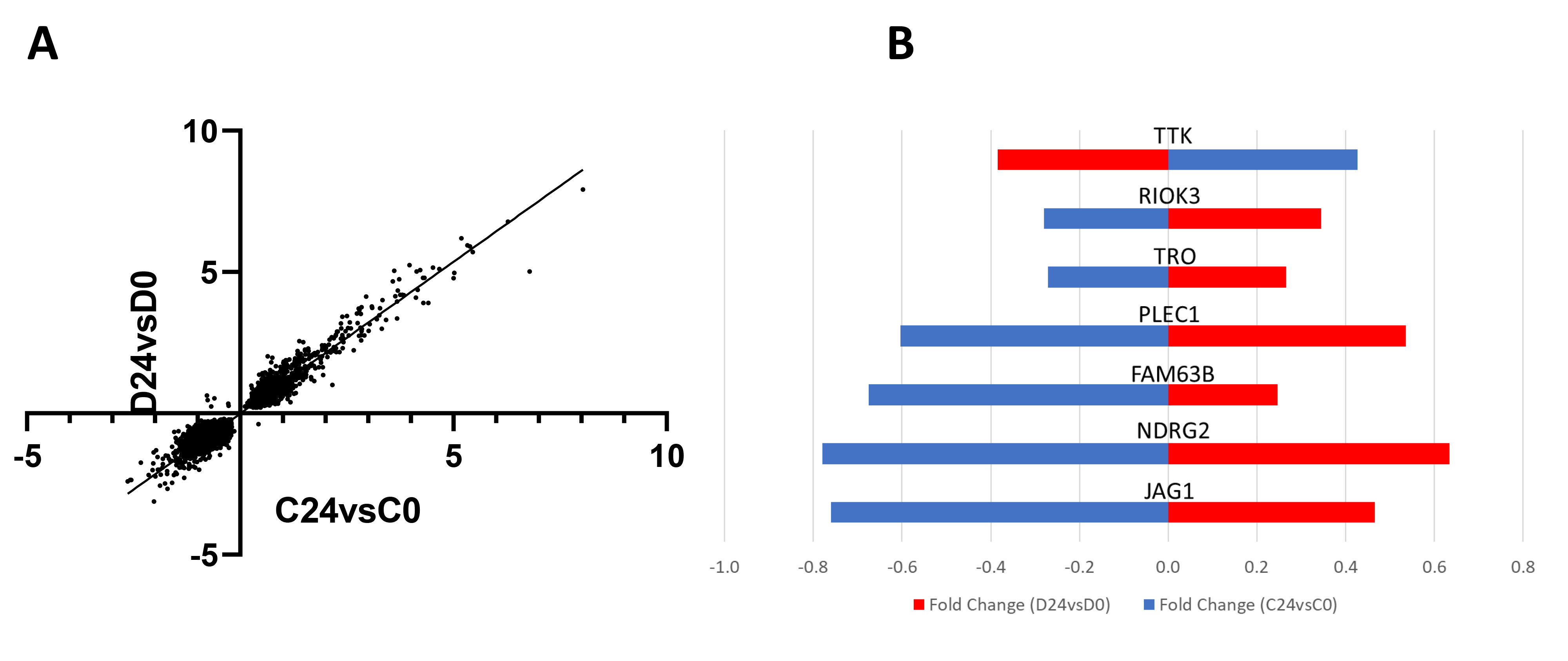

Supplement: Supplementary file 1 [file genes-12-00047-s001.zip › genes-1033129-supplementaryNEW/Suppl 1.tif]

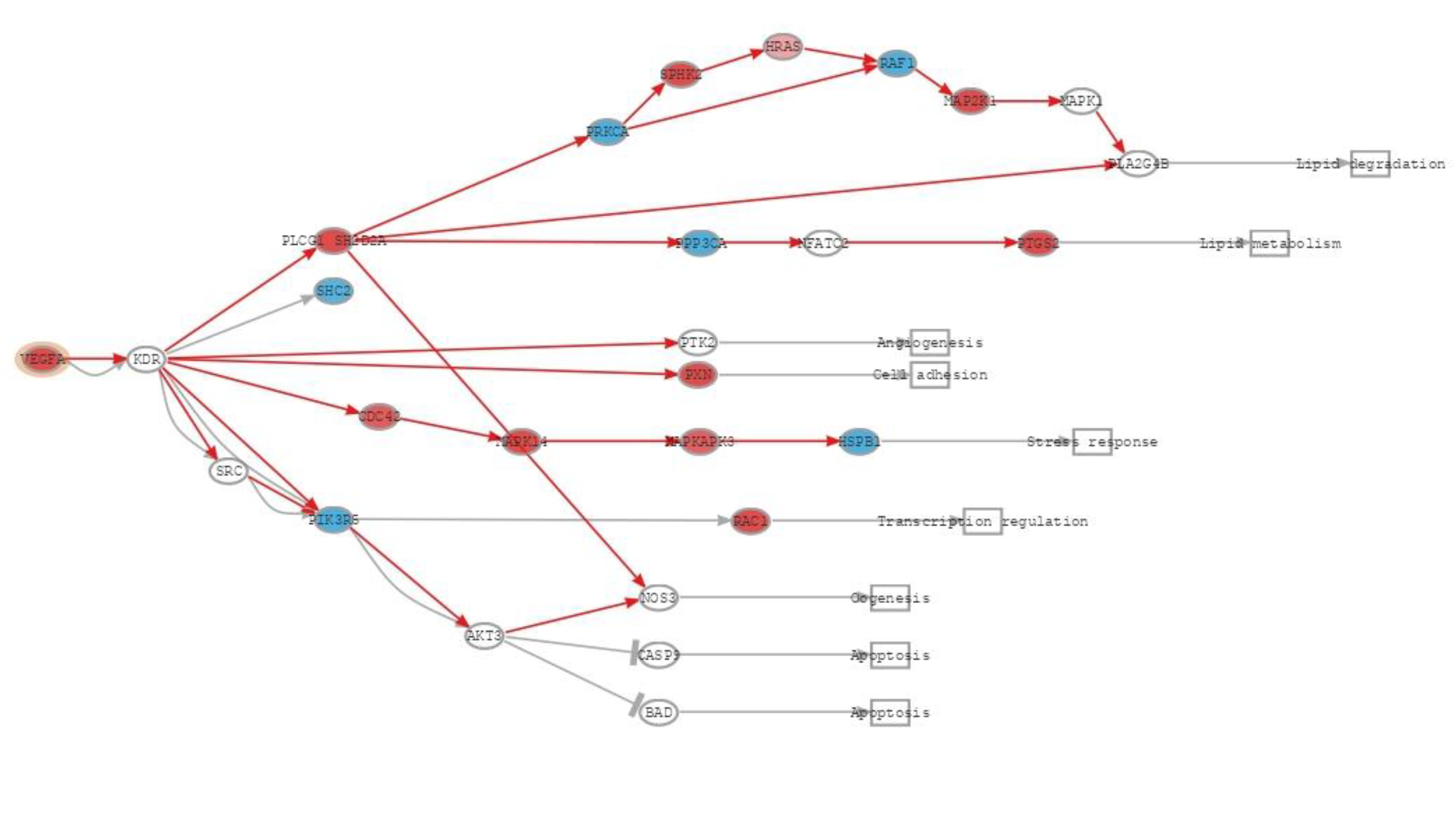

Supplement: Supplementary file 1 [file genes-12-00047-s001.zip › genes-1033129-supplementaryNEW/Suppl 2.tif]
